# New onset of Bell’s palsy in a patient with West Nile Encephalitis

**DOI:** 10.1002/ccr3.3009

**Published:** 2020-06-18

**Authors:** Tetyana Vaysman, Anna Melkonyan, Antonio Liu

**Affiliations:** ^1^ Department of Medicine University of Maryland Capital Region Health Cheverly MD USA; ^2^ Department of Neurology Adventist Health White Memorial Los Angeles CA USA

**Keywords:** Bell's palsy, encephalitis, West Nile virus

## Abstract

The case report presented a patient who was diagnosed with West Nile virus encephalitis and developed new onset of Bell's palsy within 8 days of diagnosis. Given the incidence of WNV, it would be beneficial to evaluate WNV‐infected patients for peripheral neuropathy which nowadays has quite practical implication.

## Graphical abstract

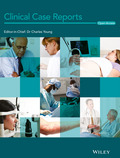

## 1 INTRODUCTION

West Nile virus (WNV) is the leading cause of mosquito‐borne disease in the United States and is most commonly spread through an infected mosquito bite even though other modes of transmission such as breastfeeding, transplacental transmission, organ transplantation, blood transfusion, and laboratory acquisition exist.^1^ The virus is a member of the Flavivirus genus, making it closely related to the viruses that cause yellow fever, dengue fever, and St Louis encephalitis.^2^ The virus was first discovered in Uganda in 1937 and first reported in North America in 1999.^3^ Since then, thousands of cases are reported each year with major outbreaks in August and September.^4^ To diagnose WNV as the underlying etiology of encephalitis, it is informative to obtain cerebrospinal fluid (CSF) for detection of WNV IgM antibody in addition to the serum antibodies.^5^

West Nile virus is the most common cause of viral encephalitis in the United States and is considered a significant contributing cause for numerous cases of neurological dysfunction in the nearest future.^6^ According to the US Center for Disease Control and Prevention, approximately 80% of infected people are asymptomatic or have mild symptoms.^7^ All ages are affected with highest incidence of death and CNS involvement among people 60‐89 years old.^8^ Risk factors embrace immune suppression, advanced age, and chronic diseases such as diabetes, chronic kidney failure, and hypertension. While most persons infected with WNV are asymptomatic, 20%‐40% of those who are affected, develop symptoms. However, the opposite end of the disease continuum introduces a severe encephalopathy and can even result in coma or death. Additionally, many patients with WNV encephalitis exhibit some movement disorders including tremor, myoclonus, and Parkinsonism.^9^ Less than 1% of affected people develop neuroinvasive disease and mortality rate accounts for <0.1% of all WNV‐infected patients.^8^

Bell's palsy is an idiopathic facial nerve paralysis which often presents with unilateral facial weakness commonly characterized by an inability to close the eye, disappearance of the nasolabial fold, and eyebrow drooping at the affected corner of the mouth. There may also be decreased tearing, hyperacusis, and/or loss of taste sensation on the anterior two thirds of the tongue.^10^ The nerve paralysis was named after Scottish physician, surgeon, and neurologist Charles Bell (1774‐1842) who first described the lesions of facial nerve (CN VII) and its clinical presentations.^11^ It has been widely accepted that herpes simplex virus and herpes zoster virus are the most common etiology of Bell's palsy. Other viral infectious agents such as Epstein‐Barr Virus, cytomegalovirus, rubella, mumps virus, adenovirus, influenza B virus, and coxsackievirus have been reported.^12^ However, there are no known reports of development of Bell's palsy with WNV encephalitis. The annual incidence of Bell's palsy is 40 000 new cases each year with 8% to 12% recurrence rate.^13^ Up to 70% of patients will have complete resolution without treatment.^14^ Even though the underlying pathophysiology remains elusive, Bell's palsy is thought to result due to virus‐mediated inflammation resulting in swelling of the CN VII and its compression at the geniculate ganglion.^12^

## 2 CASE PRESENTATION

A 71‐year‐old right‐handed female patient with history of mild dementia, obesity, hypertension, thyroid disorder, and type 2 diabetes presented to Emergency Department in early October with a gradual onset of altered mental status, headache, and intractable nausea and vomiting over the last 4‐5 days prior to admission. Despite being diagnosed with mild dementia, the patient was independent in all activities of daily living but demonstrated remarkable deviation in mental status from her baseline on admission. She denied recent travelling and sick contacts in the prior month and was not able to recall any mosquito or insect bite. Social history revealed no smoking, using alcohol, or consuming illicit drugs. Home medication list included metoprolol succinate, levothyroxine, enalapril, simvastatin, pantoprazole, metoclopramide, and insulin aspart with no known drug allergy. Vital signs on admission showed elevated temperature 102.7 F, tachycardia with heart rate 106 beats/min, hypertension with blood pressure 166/86 mm Hg, respiratory rate 16 per minute, and oxygen saturation 98% on room air. On physical examination, her neck was supple and nontender with negative Kernig and Brudzinski signs. Even though the patient appeared in no acute distress, she was overall drowsy and slow in response but denied shortness of breath, chest pain, and difficulty of breathing or any other acute distress. The patient was oriented to name and place only and was not attentive enough to do registration and recall test. Neurological examination revealed grossly intact Cranial nerves II‐XII with no facial droop or speech impairment. Motor examination showed normal tone, bulk and strength, and unaltered sensory perception to light touch and temperature (ice application). No urine or bowel incontinence was reported. Despite the patient required assistance with the ambulation, she demonstrated a steady gait; however, co‐ordination examination was deferred due to generalized weakness. Laboratory data were significant for serum white blood cell count of 4.8, hemoglobin of 13.8, and hematocrit of 41.9, platelets 120 000 with normal chemistry and coagulation studies. Initial CT of the head and MRI of the brain were negative for any acute pathology. Lumbar puncture performed and CSF analysis revealed clear appearance with opening pressure of 23 cm CSF (normal CSF opening pressure is between 18 and 20 cm CSF), white blood cells of 100 cells/µL with 98% lymphocytes (normal level is 0‐5 cells/µL), 117 mg/dL protein (normal level is less < 45 mg/dL), and 120 g/L glucose in the CSF which pointed toward the viral etiology of the presumed insulting agent.

While CSF cultures and immune studies were pending, empiric treatment with vancomycin, ceftriaxone, ampicillin, and acyclovir was initiated. All cultures and PCR results for viral agents were negative except of positive West Nile titer. CSF results revealed: WNV antibody IgM level was 6.51 (normal < 0.89), and antibody IgG level was 2.38 (normal < 1.29). Patient's condition remained stable without any changes or deterioration during the first 5 days of hospitalization, and her wakefulness and mentation were gradually improving. Daily neurological examinations were normal until day 8 when she developed mild but noticeable facial nerve palsy on the right side which progressively worsened and became obvious by day 10. While CN VII deficit remained the only neurologic concern, repeated MRI, MRA, and MRV were all negative. With initiated glucocorticoid therapy, the patient was given a high dose of oral prednisone 60 mg daily for 10 days followed by tapering dose of prednisone for next 10 days. Intravenous acyclovir with calculated dose per weight was concomitantly initiated and completed with a total length of 7 days course. Patient experienced little improvement of the CN VII deficit during the hospitalization despite her mental status has returned back to her baseline (mild dementia). Patient was evaluated by neurologist 1 year later on outpatient basis. At that time, her examination was completely nonfocal without any facial droop.

## 3 DISCUSSION

Immune system defense ability deteriorates with aging, and elderly population is affected the most.^15^ In addition, comorbid conditions contribute greatly to the decline in fighting infections and mounting an appropriate immune response.^16^ The patient's history includes multiple medical conditions such as hypertension, diabetes, and thyroid disorder which could potentially have deliberating effect on immune system in different ways. The etiology of patient's encephalopathy has been established promptly; however, whether WNV was a causative agent of peripheral neuropathy remains debatable. Since Bell's palsy is usually idiopathic in nature, clinicians have had a long discussion on whether the presentation was a coincidental idiopathic Bell's palsy or manifestation of West Nile virus infection.

Two hypotheses which can shed a light on ambiguous demonstration and the sequence of developing CN VII neuropathology for this patient have been discussed with supportive evidence. The first one is assumption that the patient's hypothyroid state, in which autoimmune‐mediated response triggers accumulation of fluid in the tissues and subsequent swelling, can cause entrapment of the cranial nerve. Many cases have been described in literature where after proper management of hypothyroid state with levothyroxine, Bell's palsy was resolved within a short period of time. Lee et al reports a case of sudden onset of Bell's palsy in 13‐year‐old boy who was treated with prednisolone and antiviral medications with no improvement but recovered rapidly with thyroxine replacement.^17^ However, our patient's hypothyroid state was well controlled with medications for long time and no changes were made in therapeutic management recently. The second assumption that side effects of home medications may play a role of a contributing factor was carefully reviewed considering patient's polypharmacy. Her home medications, such as metoprolol succinate, levothyroxine, enalapril maleate, simvastatin, pantoprazole, metoclopramide, and insulin aspart, were promptly resumed on admission. One case of acute onset of Bell's palsy in 62‐year‐old female patient was described by Ellis et al, when patient developed severe hypertension with subsequent unilateral facial weakness due to nonadherence with her antihypertensive therapy for 1 week.^18^ Our patient presented with hypertension which has been resolved with pain and fever management and could be considered as the least convincing factor contributing to the evolution of her facial paralysis. People who take metoclopramide can develop Bell's palsy as well; however, for those who take it over 5 years the probability of developing facial paralysis declines to 0%. Among patients who claimed to have side effects when taking pantoprazole, only 0.02% reported Bell's palsy. To alleviate the side effects of medication, usually an insulting agent is removed with full or partial resolution of symptoms. In our case, the patient had full recovery of facial nerve dysfunction within months but she was consistently taking pantoprazole, which argues against the side effect of this medication as a contributing factor.^19^

By weighting all plausible explanations of new onset Bell's palsy in this patient, the most feasible justification gravitates toward the WNV as a causative agent of her neuropathy. Research studies have revealed interesting findings of WNV effects on neuronal tissue. Based on investigations, WNV causes proven upregulation of class I and II major histocompatibility complex in infected human embryonic myoblasts as well as in Lewis rat Schwann cells. Such experimental evidence highlights significant implication of the virus on autoimmune response and subsequent induction of inflammatory condition.^20^ Other authors demonstrated that WNV can upregulate the cell surface expression of immune recognition sites which results in increased recognition and binding between the virus and cytotoxic T cells.^21^ Increased production of proinflammatory cytokines from the WNV‐infected cell as well as upregulation of proinflammatory genes shed a light on the cascade of immunochemical events leading to neurotoxicity. Based on these findings, it is postulated that virus‐induced inflammation is a major contributing factor to neuropathogenesis.^22^ It has been suggested that elevated level of some cytokines lead to a postinfectious proinflammatory state which can remain in the bloodstream from months to years contributing to neuroinflammation in WNV‐affected people and resulting in poor recovery.^23^ Hence, the management of Bell's palsy has been geared toward reducing the inflammation since no prevention or cures exist.

Empiric treatment with ceftriaxone, vancomycin, ampicillin, and acyclovir was initiated on admission. These medications have been reviewed carefully for side effects including neuropathies; however, they were not suspected as underlying and provocative agents. Currently, there is no specific antiviral treatment for West Nile virus infection and the mainstay of treatment is pointed toward reducing fever and pain control. In severe cases, such as in WNV encephalitis, hospitalization and supportive treatment with intravenous hydration, systemic inflammatory response management, and treatment with steroids are necessary.^8^ The recommended treatment for facial nerve palsy is oral glucocorticoids (prednisone 60‐80 mg/d for at least 1 week) within 3 days of symptom onset.^24^ Some authors speculate that treatment with corticosteroids started within 72 hours significantly improves chances for complete recovery but there is no added benefit from acyclovir.^25^ In contrast, other literature review highlights the opposite side of the debates. In a subgroup of patients with severe facial palsy (House‐Brackmann grade IV or higher), it has been found to be beneficial to introduce antivirals in addition to glucocorticoid treatment. The reasoning behind antiviral therapy is the impression that Bell's palsy is most commonly caused by herpes simplex virus.^24^ Antivirals used include valacyclovir and acyclovir, both effective agents in herpes simplex virus infection. Our patient was started on oral prednisone and intravenous acyclovir and no improvement of facial nerve palsy during the hospitalization suggested less likely herpetic etiology. WNV could be detected in serum and CSF for months to years following neuropathologic conditions and its severity greatly correlates with immune system state (immunocompetent/immunocompromised). With subsequent follow‐up visit in 1 year, the patient was found to have no facial droop and no focal neurological deficit with a complete resolution of the facial nerve palsy. This may be the first time CN VII deficit is described as a manifestation of West Nile virus encephalitis.

## 4 CONCLUSION

Our case demonstrates that viral infection can impose long lasting impairment even though serious neuroinvasive complications are rare.^26^ With no approved vaccine and limited treatment options, WNV has become the major public health concern in the United States causing recurring outbreaks for the last two decades.^27^ Merging our knowledge of the disease pathogenesis and presentation is crucial to navigate therapeutic management and to facilitate prompt treatment which may significantly improve the outcomes.

## CONFLICT OF INTEREST

The authors declare that they have no conflicts of interest regarding the publication of this paper.

## AUTHOR CONTRIBUTIONS

TV (primary author): assumed direct responsibility for the publication, working on revised versions by confirming accuracy of collected data, insuring that all deserving authors have been credited; was responsible for the literature review and final approval, submitting revisions and final version, proofreading, and communicating with editors. AM (co‐author): contributed greatly to data collection and analysis, provided guidance in manuscript drafting, data interpretation and literature review, developed draft of the case report, major contribution to introduction, and case presentation sections of the manuscript. AL (corresponding author): provided consult from Neurology Department and was responsible for supervision, guidance, and final approval of the manuscript; assumed all administrative and executive functions; and ensured the accuracy and integrity of the manuscript were appropriately investigated.
